# Overexpression of the *Salix matsudana* aquaporin gene *SmPIP1;3* enhances plant resistance to abiotic stresses

**DOI:** 10.3389/fpls.2025.1685356

**Published:** 2025-10-14

**Authors:** Xiao Xu, Peng Yin, Lei Wang, Xiaoxiong Lu, Jichen Xu

**Affiliations:** ^1^ Coastal Salinity Tolerant Grass Engineering and Research Center, Ludong University, Yantai, China; ^2^ State Key Laboratory of Tree Genetics and Breeding, College of Biological Sciences and Technology, Beijing Forestry University, Beijing, China

**Keywords:** plasma membrane intrinsic proteins (PIPs), *Salix matsudana*, SmPIP1;3, overexpression, abiotic stress tolerance

## Abstract

Plasma membrane intrinsic proteins (PIPs) are involved in plant growth and stress adaptation through their dynamic gating mechanism. Nevertheless, pinpointing the specific roles of individual isoforms remains challenging because of their functional pleiotropy and integrated responses to diverse cues. In this study, we characterized a salt-responsive aquaporin gene, designated *SmPIP1;3*, isolated from a salt-resistant *Salix matsudana* variety. Bioinformatics analysis confirmed that it encodes a protein that possesses canonical PIP features with six transmembrane domains, five interhelical loops, and seven serine phosphorylation sites involved in phosphorylation-mediated regulation. The *SmPIP1;3* gene was introduced in tobacco plants, and its heterologous expression conferred significant morphological improvements, including taller plant height, larger leaves, longer roots, and increased biomass. Under salt, drought, cold, and heat stresses, transgenic plants showed substantially alleviated membrane damage, as evidenced by weakened Evans blue staining. Consistently, their malondialdehyde contents were 1.48-, 1.47-, 1.57-, and 1.62-fold lower, while relative electrolyte leakage values were 1.56-, 1.35-, 1.53-, and 1.61-fold lower than those of wild-type plants, respectively. *SmPIP1;3* orchestrates multi-stress tolerance by sustaining physiological homeostasis and limiting membrane damage. Its performance positions it as a valuable genetic asset for molecular breeding programs.

## Introduction

Water uptake and transmembrane water transport are fundamental physiological processes underlying plant growth and development and also serve as a crucial regulatory pathway for plant adaptation to abiotic stress (Gill et al., 2021). Water movement across cellular membranes is a well-established, osmotic-driven process. Aquaporins (AQPs), specialized water-channel proteins located in biological membranes, selectively facilitate water flux while largely preventing proton leakage and, in some cases, allowing the passage of small solutes such as glycerol or CO_2_ (Eisenbarth and Weig, 2005; Sohail et al., 2022). Based on the phylogenetic relationship and subcellular localization, AQPs in higher plants can be classified into five subfamilies: plasma membrane intrinsic proteins (PIPs), tonoplast intrinsic proteins (TIPs), nodulin 26-like intrinsic proteins (NIPs), small basic intrinsic proteins (SIPs), and X-intrinsic proteins (XIPs) (Kaldenhoff and Fischer, 2006). Among these, PIPs are among the most extensively studied groups, exhibiting high sequence conservation, and can be systematically classified into two phylogenetic clades, designated as PIP1s and PIP2s (Törnroth-Horsefield et al., 2006).

Abiotic stress factors, such as drought, salinity, cold, and heavy metals, can disrupt a plant’s water homeostasis, leading to severe dehydration and death, thereby limiting plant growth, development, and productivity (Suslov et al., 2024). Recent studies have highlighted the functional versatility of AQPs, particularly PIPs, which exhibit bidirectional water transport driven by transmembrane osmotic gradients, with dynamic gating mechanisms being regulated by phosphorylation and osmotic stimuli (Horie et al., 2011). The expression patterns of PIPs vary substantially across various plant organs (e.g., roots and leaves) and along different durations of stress (Horie et al., 2011). For example, a study focused on *Arabidopsis* found that most PIP genes were downregulated in the leaves under drought, except for *AtPIP1;4* and *AtPIP2;5*, which were upregulated (Alexandersson et al., 2005). The overexpression of *AtPIP1;4* or *AtPIP2;5* in *Arabidopsis* also improved resistance to cold stress (Rahman et al., 2020). The *GoPIP1* gene identified from *Galega orientalis* was significantly induced by 200 mmol/L NaCl or 20% polyethylene glycol (PEG) 6000 treatment. The overexpression of this gene in *Arabidopsis* increased vulnerability to drought stress but not to salinity (Li et al., 2015). Transgenic tobacco expressing *BnPIP1* from *Brassica napus* exhibited enhanced drought tolerance, improved growth, and higher seed germination rates, whereas antisense plants demonstrated developmental defects and heightened drought susceptibility (Yu et al., 2005). The overexpression of *OsPIP1;1*, a salt-inducible PIP gene from rice, conferred improved resistance to salinity in *Arabidopsis* (Guo et al., 2006). Moreover, transgenic bananas overexpressing *MusaPIP1;2* demonstrated enhanced tolerance to cold, salinity, and drought compared to wild-type (WT) plants (Sreedharan et al., 2013). While substantial experimental evidence underscores the roles of PIP aquaporins in stress responses, elucidating the precise contributions of individual genes or isoforms is still difficult owing to their functional pleiotropy and integrated regulation under different environmental stimuli (Afzal et al., 2016). Consequently, systematic characterization of PIP family members is imperative to decipher the molecular basis of plant stress resilience.

*Salix matsudana* is an important afforestation and industrial timber tree species. A previous transcriptome analysis revealed that the expression of *SmPIP1;3* was induced by salt stress in the salt-resistance 9901 variety of *S. matsudana* (Wang et al., 2024). In this study, we aimed to characterize the *SmPIP1;3* gene to elucidate its evolutionary attributes and to assess its functional roles in plant growth and environmental stress resistance through genetic transformation. Our findings enrich the existing understanding of the stress resistance process in willow trees and offer a valuable gene resource for future molecular breeding strategies.

## Materials and methods

### Plant materials and treatment

Cuttings of *Salix mandshurica* 9901 were cultivated in pots (20 cm × 20 cm) filled with sandy soil in a growth chamber (2,000 lux light intensity, a 16-hour light/8-hour dark cycle, 70% relative humidity, 25°С). Plants were irrigated with water once every 4 days and with Hoagland solution (Hoagland and Arnon, 1950) once a week. After 1 month, they were irrigated with 300 mL of 200 mmol/L NaCl solution per pot. Leaves and roots were harvested at 0 and 24 h after salt application for RNA extraction.

### Expression assessment of *SmPIP1;3* in willow

The total RNAs were extracted from the leaves and root samples using the polysaccharide polyphenol kit (Tiangen, Beijing). Then, RNA was transcribed into cDNA using the Hifair^®^III 1st Strand cDNA Synthesis SuperMix for polymerase chain reaction (PCR) kit (Yeasen Biotech, Shanghai, China). Based on the genome sequence data of *S. mandshurica*, the specific primers were designed for *SmPIP1;3* gene fragment (SmPIP1;3F, 5′-TGGCCTTGGTGCTGAGATAA-3′; SmPIP1;3R, 5′-CTCCTTGCTGGGTTAATGCC-3′). The qRT-PCR assay was conducted for expression test with the following procedure: 95°C for 5 min, 40 cycles of 95°C for 10 s, and 58°C for 30 s. *Ubiquitin* (*UBQ*) was used as the reference gene (UBQF, 5′-AGAAGGAGTCAGCAACGATG-3′; UBQR, 5′-CATTAGGTTCTGAACAGCAGG-3′).

### Gene cloning of *SmPIP1;3*

Based on the genome data of *S. mandshurica*, the specific primers were designed at both sides of *SmPIP1;3* gene fragment with additional restriction enzyme digestion sites (SmPIP1;3HindIIIF, 5′-cccaagcttATGGAAGGCAAAGAAGAGGA-3′, the lowercase letters mean *Hin*dIII digestion bases; SmPIP1;3XbaIR, 5′-tgctctagaTTAAGCTCTGCTCTTGAAAG-3′; the lowercase letters mean *Xba*I digestion bases). The full-length gene fragment was amplified from the cDNA sample of 9901 willow at 4-day salt treatment and ligated to the pGEM-T easy vector (Promega, Madison, WI, USA) following the manufacturer’s instructions. The recombinant plasmid was transformed into the bacterial strain TOP10, and the positive strain was used for plasmid sequencing.

### Sequence characterization of *SmPIP1;3*

The PIP1 sequences in *Populus trichocarpa* v3.1 genome were obtained (https://phytozome-next.jgi.doe.gov/info/Ptrichocarpa_v3_1), and homologs of PIP1;3 from seven plant species (*P. trichocarpa*, *S. matsudana*, *Hevea brasiliensis*, *Solanum lycopersicum*, *Vitis vinifera*, *Oryza sativa*, and *Zea mays*) were obtained from the NCBI database (https://www.ncbi.nlm.nih.gov/). Multiple sequence alignment was conducted using the DNAMAN v7.0 software. The phylogenetic tree was performed using MEGA7 with the bootstrap value set at 1,000 repetitions. The online software ProtParam (https://web.expasy.org/protparam/) was used to analyze the physical and chemical properties of SmPIP1;3 proteins. Transmembrane helices were predicted using TMHMM Server v 2.0 (http://www.cbs.dtu.dk/services/TMHMM/).

### *SmPIP1;3* gene transformation to tobacco plants

The recombinant plasmids and gene expression vector pEZR(K)-LC were digested with restriction enzymes *Hin*dIII and *Xba*I and ligated to form the recombinant expression plasmids. The resulting constructs were introduced into *Agrobacterium tumefaciens* strain GV3101 using the electroporation method. The positive strain was identified and cultured in Yeast Extract Broth (YEB) medium containing 50 mg/L kanamycin and 10 mg/L rifampicin at 28 °C overnight and then transferred to antibiotic-free YEB liquid medium until OD600 0.6–0.8. The healthy tobacco (*Nicotiana tabacum* L.) leaves were cut and immersed in the bacterial solution for 10 min, transferred to the co-cultivation medium [Murashige and Skoog (MS) medium supplemented with 2 mg/L 6-benzylaminopurine (6-BA), 0.2 mg/L 1-naphthaleneacetic acid (NAA), 3% sucrose, and 0.55% agar] in darkness for 3 days, and transferred to the screening medium (MS with 2 mg/L 6-BA, 0.2 mg/L NAA, 3% sucrose, 0.55% agar, 100 mg/L kanamycin, and 200 mg/L timentin) in light for 30 days. The regenerated buds were transferred to the rooting medium (MS with 3% sucrose, 0.55% agar, 50 mg/L kanamycin, and 200 mg/L timentin) for 20 days to obtain the transgenic seedlings.

Genomic DNA was extracted from the leaves of regenerated plants and screened by PCR tests using the specific primer pair SmPIP1;3HindIIIF/SmPIP1;3XbaIR. Further, the total RNAs were extracted from the DNA-positive lines, reverse-transcribed into cDNA, and assayed for *SmPIP1;3* gene expression by RT-PCR. The lines with the overexpression of *SmPIP1;3* were recognized as transgenic and used for subsequent assays.

### Physiological index tests of tobacco plants

The transgenic and WT tobacco plants were propagated by stem cutting. Seedlings were transplanted into pots (10 cm × 10 cm) filled with vermiculite and maintained in the growth chamber (2,000 lux light intensity, a 16-hour light/8-hour dark cycle, 70% relative humidity, 25°С) for 1 month. The plants were irrigated with water every 3 days and supplied with Hoagland solution once a week. Salt treatment was conducted with 150 mL NaCl solution (200 mmol/L) per pot. Leaves were harvested after 0- and 4-day treatments for physiological index tests. Three replicates for each line/treatment were conducted. Statistical analyses were performed using the SPSS 23.0 software. One-way ANOVA followed by Duncan’s multiple-range test was applied, * and ** indicate significant differences at p < 0.05 and p < 0.01, respectively.

Relative electrolyte leakage (REL): 0.1 g fresh leaves were cut into small pieces (0.5 cm × 0.5 cm), soaked in 30 mL deionized water, and shaken at 25 °C at 180 rpm overnight. The electrical conductivity was measured as R1 using the electrical conductivity meter (DDS-11C). The solutions with the samples were then autoclaved at 121°C for 20 min, cooled naturally to room temperature, and then re-measured as R2. Relative conductivity was calculated as R1/R2.

Malondialdehyde content (MDA): 0.1 g of fresh leaves were homogenized in 1 mL of 10% trichloroacetic acid (TCA). The homogenate was centrifuged at 12,000 rpm for 5 min. The supernatant was collected, mixed with 1 mL of 0.6% thiobarbituric acid (TBA), and boiled for 15 min. After centrifugation at 12,000 rpm for 5 min, the supernatant was collected for absorbance measurement at 450, 532, and 600 nm. MDA content was calculated as follows: [6.45 × (OD532 − OD600) − 0.56 × OD450] × total extract volume/fresh weight of sample.

## Results

### Expression pattern of *SmPIP1;3* in *S. matsudana*

**Figure 1:**
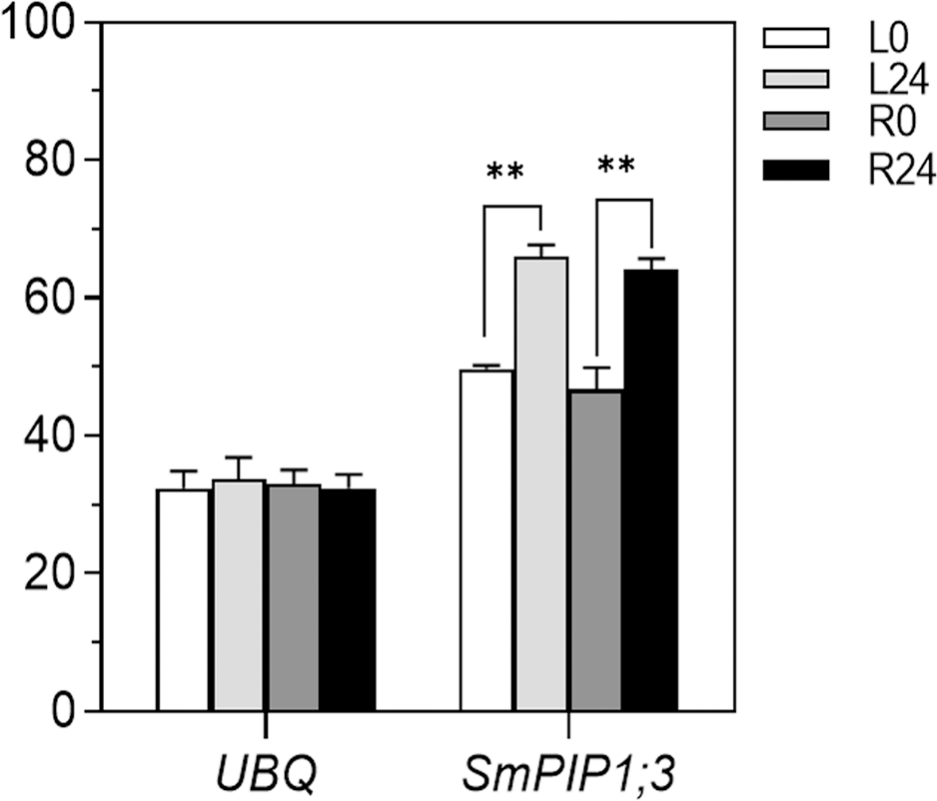
Expression pattern of *SmPIP1;3* in 9901 willow under salt treatment. *UBQ*, ubiquitin gene used as an internal reference for normalization; L0 and L24, leaf samples at 0 and 24 h, respectively, of salt treatment; R0 and R24, root samples at 0 and 24 h, respectively, of salt treatment. ** indicates a statistically significant difference (p < 0.01) between 0 and 24 h of salt treatment.

The expression pattern of *SmPIP1;3* in the salt-resistant *S. matsudana* 9901 variety was examined using gene-specific primers SmPIP1;3-F/SmPIP1;3-R (**Figure 1**). *SmPIP1;3* transcripts were expressed at comparable levels in the leaves and roots under control conditions. When subjected to salt treatment, the *SmPIP1;3* transcript abundance significantly increased in both leaf and root tissues by more than 1.30- and 1.33-fold, respectively. This indicates that *SmPIP1;3* may be involved in resisting salt stress in *S. matsudana*.

### Characterization of the properties of *SmPIP1;3*

**Figure 2:**
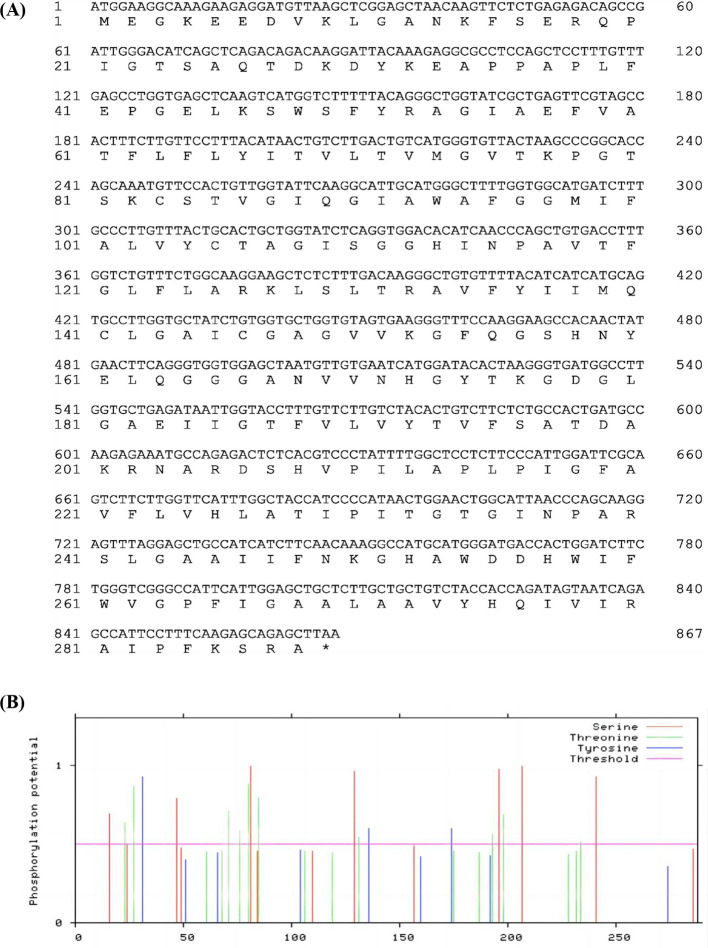
*SmPIP1;3* nucleotide and amino acid sequence **(A)** and phosphorylation amino acids **(B)**. The x-axis represents the amino acid order.

The full-length fragment of *SmPIP1;3* was amplified and cloned from the 9901 cDNA sample with the SmPIP1;3HindIIIF/SmPIP1;3XbaIR primers. The sequencing results revealed a length of 867 bp, encoding 288 amino acids (**Figure 2A**). Amino acid composition analysis showed that Gly, Ala, and Ile were abundantly present in SmPIP1;3 in proportions of 12.2%, 11.8%, and 8.7%, respectively. The two amino acids that are critical for PIP function, Ser and His, were present in proportions of 4.5% and 2.8%, respectively. SmPIP1;3 had a predicted molecular weight of 30.73 kDa, an isoelectric point of 8.99, an aliphatic index of 96.25, a grand average of hydropathicity of 0.374, and an instability index of 25.07. These parameters indicate that SmPIP1;3 is a basic, moderately hydrophobic, thermostable, and overall stable protein.

Phosphorylation is a gating mechanism that regulates the plant plasma membrane AQP activity. In SmPIP1;3, 19 putative phosphorylation sites were predicted (**Figure 2B**), including seven Ser (S) residues (S16, S49, S81, S129, S196, S207, and S241), three Tyr (Y) residues (Y31, Y136, and Y147), and nine Thr (T) residues (T23, T27, T71, T76, T85, T131, T193, T198, and T234), spreading in the protein sequence randomly.

**Figure 3:**
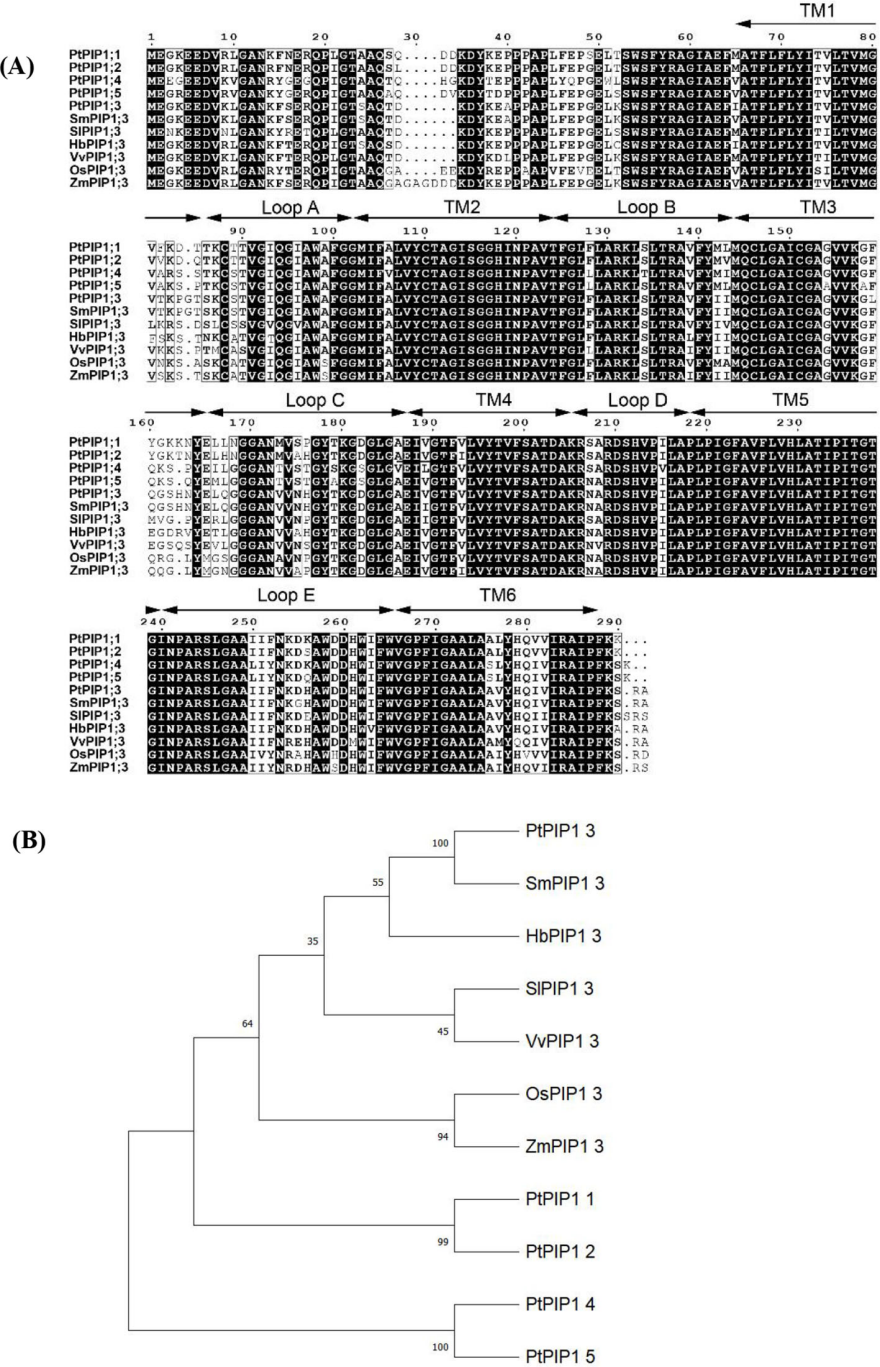
Sequence alignment of plasma membrane intrinsic proteins (PIPs) from seven species **(A)** and phylogenetic tree **(B)**. Note: *Pt*, *Populus trichocarpa*; *Sm*, *Salix matsudana*; *Sl*, *Solanum lycopersicum*; *Hb*, *Hevea brasiliensis*; *Vv*, *Vitis vinifera*; *Os*, *Oryza sativa*; *Zm*, *Zea mays*.

Further sequence alignment revealed a high level of conservation among the PIPs. SmPIP1;3 showed the highest sequence identity (98.61%) with PtPIP1;3 (**Figure 3A**). Several amino acids were revealed to be specific to the Salicaceae family, such as T82, P84, and G85, or the dicot family, such as D28, A100, E165, F252, D259, and I281. PIP1;3 thus exhibited evolutionary characteristics, and it could be used for species classification.

Based on protein sequences from seven species, a phylogenetic tree was constructed (**Figure 3B**). SmPIP1;3 was found to cluster closely with PtPIP1;3, and they are both members of the Salicaceae family but are far from the other family species. The PIP1;3 proteins from the dicot species were grouped further from those of monocot species, such as maize and rice. All PIP1;3 proteins were grouped and separated from other PIP1 subfamily members, such as PIP1;1, PIP1;2, PIP1;4, and PIP1;5, indicating that each PIP1 subfamily has its own evolutionary identity.

**Figure 4:**
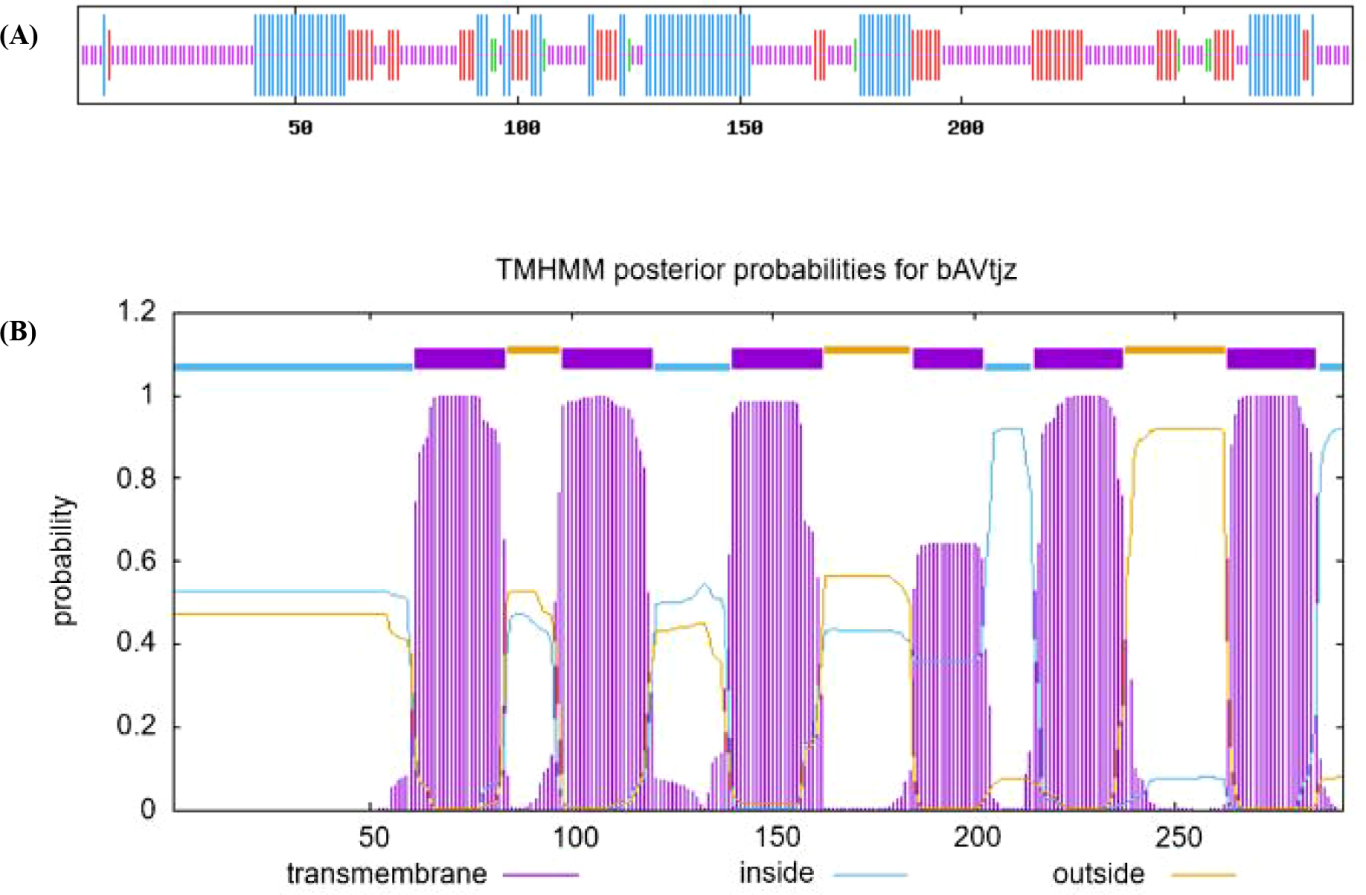
SmPIP1;3 protein secondary structure **(A)** and transmembrane structural domain **(B)**.

The secondary protein structure prediction showed that SmPIP1;3 mainly included random coils in the proportion of 48.61%, while α-helix, extended chain, and β-bend were in the ratios of 28.82%, 19.79%, and 2.78%, respectively (**Figure 4A**). Further protein domain analysis revealed SmPIP1;3 as a typical PIP1 aquaporin, containing six transmembrane domains (TM1–TM6) and five interhelical loops (Loop A–Loop E) (**Figure 4B**). Each α-helix was associated with a membrane-intrinsic connecting loop and a membrane-extrinsic connecting loop on both sides to form an hourglass and a rotating symmetric structure. The transmembrane α-helices were not parallelly arranged but staggered at an angle between 25° and 40°, forming a structure connecting the cells inside and outside.

### Effect of *SmPIP1;3* on plant growth

**Figure 5:**
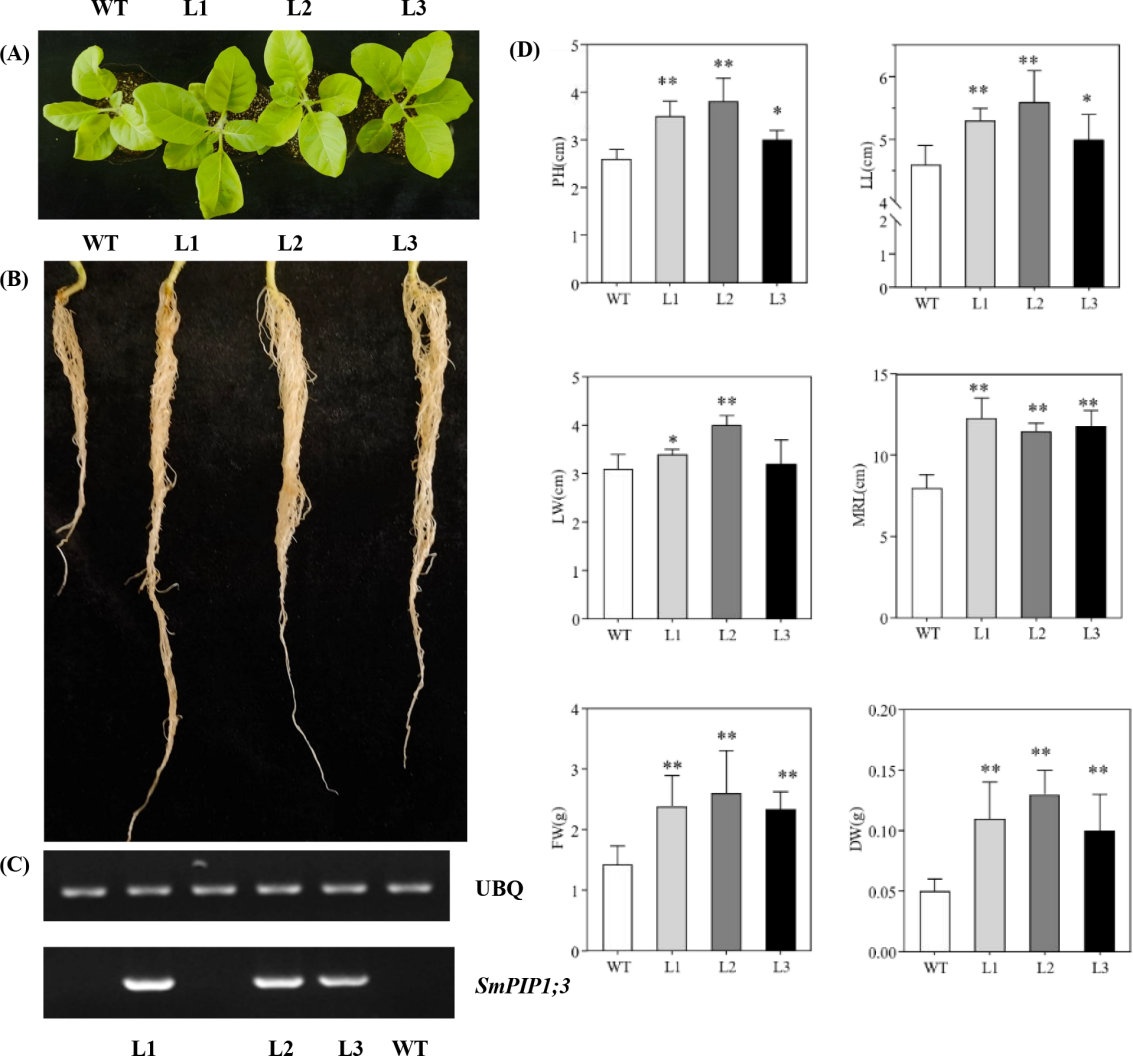
Growth performance of *SmPIP1;3-*transgenic lines (L1, L2, and L3) and wild-type plants (WT). **(A)** One-month-old tobacco leaves. **(B)** One-month-old tobacco roots. **(C)** RT-PCR detection of *SmPIP1;3* in transgenic lines. **(D)** Growth indexes. PH, plant height; LL, leaf length; LW, leaf width; MRL, maximum root length; FW, fresh weight; DW, dry weight. The data were obtained in three replicates. * and ** mean the significant difference between the transgenic lines and WT at p < 0.05 and p < 0.01, respectively.

*SmPIP1;3* was successively introduced in tobacco plants, and three independent *SmPIP1;3*-overexpressed tobacco lines (L1, L2, and L3) were obtained and further propagated (**Figure 5C**). One-month-old seedlings were used for the growth index evaluation (**Figures 5A, B**). Under normal conditions, the transgenic lines had a height between 3.0 and 3.8 cm, which was significantly higher than that of WT (2.6 cm). Meanwhile, the transgenic plants’ leaf length ranged from 5.0 to 5.6 cm and leaf width from 3.2 to 4.0 cm—an average increase of 1.15- and 1.16-fold compared to WT, respectively. The root length of the transgenic plants ranged from 11.5 to 12.3 cm—an increase of 1.44- to 1.54-fold over WT. The number of roots, however, was similar in both transgenic plants and WT. In addition, the average fresh weight and dry weight of the transgenic plants were 1.43 and 0.05 g, respectively—1.70- and 2.30-fold increase over WT, respectively (**Figure 5D**). Collectively, these results demonstrate that *SmPIP1;3* overexpression significantly improved plant growth.

### *SmPIP1;3* enhanced plant resistance to abiotic stresses

**Figure 6:**
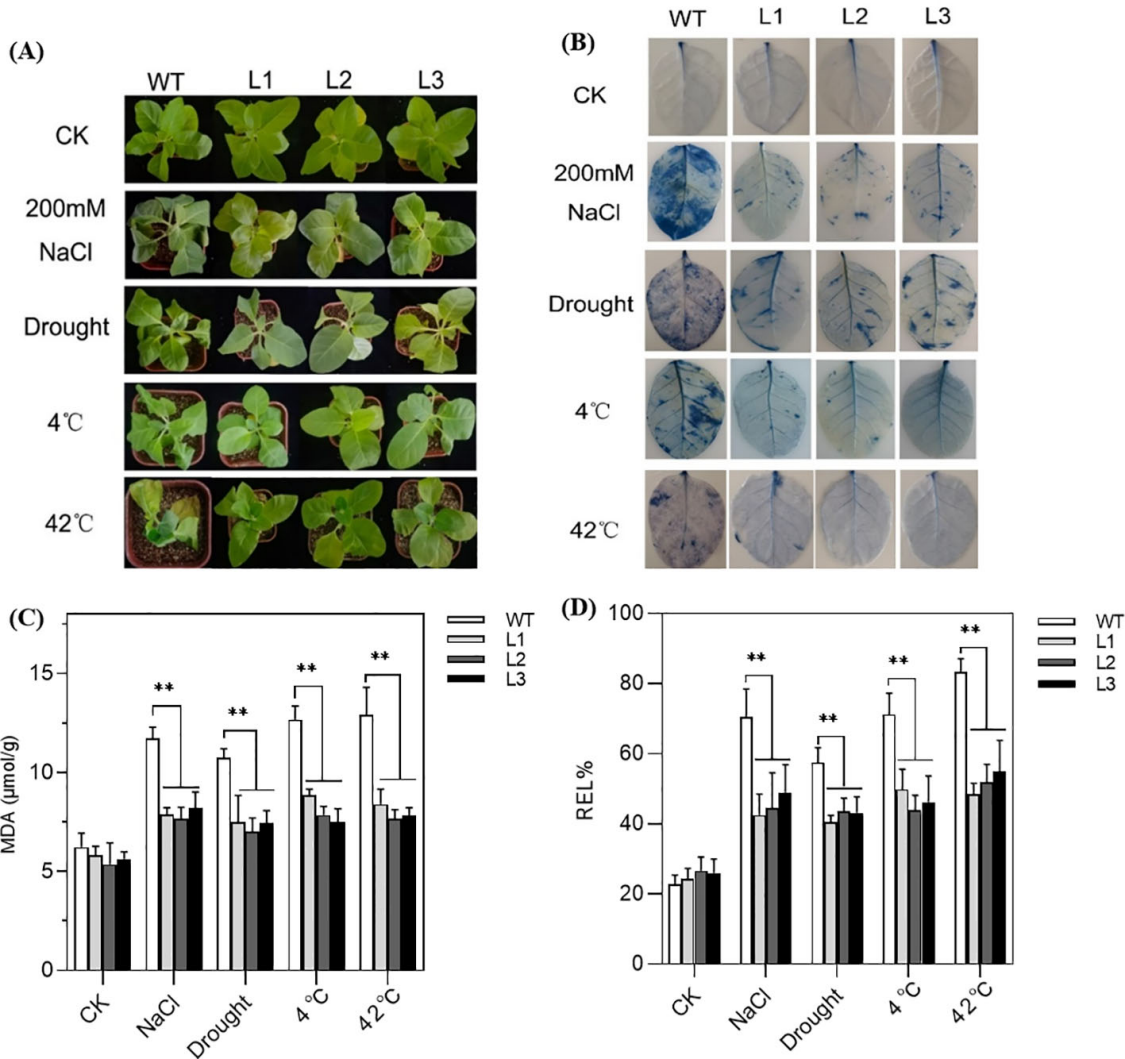
Performance of *SmPIP1;3*-overexpressing tobacco lines (L1–L3) and wild-type (WT) plants grown under control conditions or exposed to abiotic stresses: 200 mmol/L NaCl for 6 days, drought for 6 days, 4°C for 12 h, or 42°C for 4 days. **(A)** Morphological performance. **(B)** Evans blue dyeing. **(C)** Malondialdehyde content (MDA). **(D)** Relative electrolyte leakage (REL). ** mean the significant difference between the transgenic tobacco lines and WT at p < 0.01.

Under normal growth conditions, both transgenic and WT tobacco seedlings performed well and showed no obvious morphological abnormalities. When exposed to stress, the WT tobacco plants manifested severe symptoms, including wilted leaves, chlorosis, and turgor loss, while the transgenic plants fared better (**Figure 6A**). Moreover, the Evans blue staining assays showed no background signal under control conditions; however, after being exposed to stress conditions, a few interspersed blue spots appeared (**Figure 6B**). Compared with WT, the transgenic lines had less stained dye, especially under salt treatment, indicating reduced cell damage.

Physiological measurements further supported these observations. While no significant differences were detected between transgenic and WT plants under normal conditions, clear differences emerged under stress. Of them, MDA content in the transgenic plants was reduced by 1.48-, 1.47-, 1.57-, and 1.62-fold than WT under salt, drought, cold, and heat treatment, respectively (**Figure 6C**). Similarly, REL values of the transgenic plants showed decreases of 1.56-, 1.35-, 1.53-, and 1.61-fold compared with WT under the same stress (**Figure 6D**). Evidently, the overexpression of *SmPIP1;3* effectively improves plants’ physiological state and enhances their tolerance to various stresses.

## Discussion

### PIPs are widely involved in environmental stress resistance

As pivotal regulators of transmembrane water flux, PIPs play crucial roles in plant adaptation to environmental challenges. The members of this aquaporin family not only significantly enhance bidirectional membrane water permeability but also facilitate the transport of glycerol, urea, metalloids, CO_2_, and H_2_O_2_, maintaining water homeostasis, thereby establishing a fundamental adaptive mechanism for abiotic stress tolerance (Gautam and Pandey, 2021; Kumar et al., 2018). Abiotic stresses such as salinity, drought, and cold exposure differentially modulate PIP gene expression profiles. In *Arabidopsis thaliana*, most PIPs exhibited downregulated expression, except for *AtPIP1;3*, *AtPIP1;4*, *AtPIP2;1*, and *AtPIP2;5*, whose expression increased after 4 h or 2 days of drought treatment (0.25 mol/L mannitol) (Jang et al., 2004). In *Z. mays*, salt stress induced a general downregulation of ZmPIP1 and ZmPIP2, while *ZmPIP1;1*, *ZmPIP1;5*, and *ZmPIP2;4* exhibited transient upregulation patterns (Zhu et al., 2005). In *S. lycopersicum*, coordinated upregulation of *SlPIP1;1*, *SlPIP1;3*, *SlPIP1;7*, *SlPIP2;10*, and *SlPIP2;12* was observed during salt stress and the recovery phase, implying the critical role of PIPs in regulating water transport (Jia et al., 2020). In *O. sativa*, the expression level of *OsPIP1–3* was upregulated in response to 150 mmol/L salt treatment (Abdelkader et al., 2012). Similarly, this study observed significant induction of *SmPIP1;3* transcripts in both leaf and root tissues of the salt-tolerant willow species following 200 mmol/L salt stress. These stress-induced modulation patterns of PIP isoforms indicate their adaptive regulation mechanisms for preserving water balance during abiotic stress.

Comprehensive functional studies employing transgenic approaches, including both overexpression and loss-of-function mutant analyses, have provided compelling evidence for the pivotal role of PIPs in mediating plant responses to abiotic stress (Kumar et al., 2018). For example, *SpPIP1* is a drought-induced gene in *Stipa purpurea*, and its ectopic expression in *Arabidopsis* bestowed plants with superior drought tolerance as evidenced by longer roots, improved photosynthetic efficiency, increased survival rates, elevated leaf Relative Water Content (RWC), and reduced electrolyte leakage (Chen et al., 2018). Similarly, *VfPIP1*-overexpressing transgenic *Arabidopsis* plants exhibited a faster growth rate and enhanced drought resistance by reducing transpiration rates through stomatal closure (Cui et al., 2008). The overexpression of *EuPIP1;2* conferred significant drought and salinity tolerance in transgenic tobacco, manifesting as superior germination, root development, and survival rates, which were mechanistically linked to reduced oxidative membrane damage and improved osmotic adjustment. In this study, the overexpression of *SmPIP1;3* in transgenic tobacco lines enhanced growth under normal conditions, including taller plant height, larger leaves, longer roots, and increased biomass when compared with the wild type. Notably, these lines demonstrated robust multi-stress tolerance when subjected to salt, drought, cold, and heat challenges, as quantified by substantially reduced membrane damage indicators compared to wild-type controls.

These findings highlight PIP aquaporins’ functions as master regulators of abiotic stress responses, acting through mechanisms such as maintenance of membrane integrity, facilitation of osmotic adjustment, and promotion of stress-resilient growth. Further investigations are required to elucidate the regulatory networks and signaling pathways by which PIPs mediate these adaptive functions.

### *SmPIP1;3* displays pleiotropism against various stresses

Plant abiotic stress tolerance relies on complex molecular networks in which different genes contribute through distinct mechanisms. For example, expansin genes have been demonstrated to relax the cell wall components and release stress. The overexpression of the *S. matsudana* expansin gene *SmEXPA23* in tobacco decreased REL and MDA contents by 9.87% and 19.26%, respectively, compared with WT plants under salt treatment (Yang et al., 2023). Similarly, DNA damage repair tolerance (DRT) genes can promote the repair of stress-induced DNA damage, thus maintaining cell viability. The overexpression of *ZsDRT102* from *Zelkova schneideriana* significantly improved the plants’ chilling resistance, with MDA and REL levels decreasing by 10.3%–26.2% and 24.5%–35.3%, respectively, in transgenic plants relative to WT (Wang et al., 2025). PIPs constitute an efficient gene resource that can facilitate stress resistance by improving the water channel of the cell membrane (Maurel et al., 2008). In this study, the overexpression of *SmPIP1;3* in tobacco alleviated stress-induced injury by salt, drought, cold, or heat stress: the REL values declined by 1.56-, 1.35-, 1.53-, and 1.61-fold, and the MDA content fell by 1.48-, 1.47-, 1.57-, and 1.62-fold compared with WT, together with reduced Evans blue staining, indicating diminished cell damage.

Although pleiotropic genes are common, their stress spectra and directions differ markedly. *PttEXPA8* from *Populus tomentosa* was introduced into tobacco plants, and it improved the plants’ resistance to drought, heat, and cold; however, it had no significant contribution to salt and Cd stress resistance (Zhang et al., 2019). Cotton *GhWRKY25* elevated salt tolerance but reduced drought resistance (Liu et al., 2016). Moreover, the overexpression of *AtbZIP60* in tobacco enhanced its salt tolerance (Tang et al., 2012), but the overexpression of wheat *TabZIP6* and tea *CsbZIP6* in *Arabidopsis* lowered the plants’ tolerance to cold (Wang et al., 2017), thus suggesting that the homologous genes play different roles in different stress conditions. *SmPIP1;3* had a broad-spectrum resistance to all four tested stresses, making it a more versatile candidate for molecular breeding. The synergistic potential of these multi-mechanism genes warrants further exploration.

### PIPs’ function depends on their sequences and structures

Numerous studies have indicated that AQP activity is modulated by phosphorylation (Chaumont et al., 2005), which may affect AQP’s trafficking through the secretory pathway to the plasma membrane or the opening and closing of pores (Törnroth-Horsefield et al., 2006). In particular, the phosphorylation at Ser residues within the N- or C-terminal regions of PIP was more closely examined and recognized as crucial to the protein function. For example, phosphorylation at S115, S188, and S274 of SoPIP2;1 promoted the expansion of water-conducting pores in spinach (Johansson et al., 1996). In wheat, TaPIP2;10 phosphorylated at S280 facilitated CO_2_ transport into the cells, thereby resulting in enhanced photosynthesis and increasing yield, whereas the same protein phosphorylated at S121 was related to H_2_O_2_ transport into the cytoplasm (Lu et al., 2022). Several mutant assays have also confirmed the role of serine phosphorylation. The substitution of S115E in SoPIP2;1 led to a half-turn extension of transmembrane helix 1, thereby disrupting the divalent cation binding site in the gating mechanism (Nyblom et al., 2009). By contrast, the S188E mutant increased the water transport activity and supported the contribution of S188 phosphorylation to open channel formation. In our study, 19 phosphorylation sites in SmPIP1;3—seven Ser, three Tyr, and nine Thr—were determined. A sequence alignment among *SmPIP1;3* and the PIPs from seven species revealed that most of them were conserved. A previous study found that the homologous mutations of S129 and S207 in *ZmPIP2;1*, S126A, and S203A did not affect the targeting of the plasma membrane but decreased its activity by 30% to 50% (Wilder et al., 2008). Further exploration of the changes in amino acid phosphorylation in SmPIP1;3 is necessary to better understand how the water channel activity responds to various growth conditions.

The loop feature was also recognized as an important factor for the functioning of the PIP channel. One study revealed that loop C residues contribute to the selectivity of solute transport (Wilder et al., 2008). We compared the residues of the PIP1 proteins and found that loop C contained a large number of Gly residues (6–8), indicating that these PIP1s are much more flexible members (Casino et al., 2014). The sequence alignment of PIP1;3 among the seven species also demonstrated more polymorphism in loop C than the others. These performances may support the diversity of PIP1;3 against various environmental stresses, like the transported solutes. More research attention on loop C and the other domains is essential for future gene modification and molecular breeding programs.

## Data Availability

The original contributions presented in the study are included in the article/Supplementary Material. Further inquiries can be directed to the corresponding author.

## References

[B1] Abdelkader A. F. El-Khawas S. El-Din El-Sherif N. A. S. Hassanein R. A. Emam M. A. Hassan R. E. S. (2012). Expression of aquaporin gene (*OsPIP1-3*) in salt-stressed rice (*Oryza sativa* L.) plants pre-treated with the neurotransmitter (dopamine). Plant Omics 5, 532–541.

[B2] Afzal Z. Howton T. C. Sun Y. Mukhtar M. S. (2016). The roles of aquaporins in plant stress responses. J. Dev. Biol. 4, 9. doi: 10.3390/jdb4010009, PMID: 29615577 PMC5831814

[B3] Alexandersson E. Fraysse L. Sjövall-Larsen S. Gustavsson S. Fellert M. Karlsson M.. (2005). Whole gene family expression and drought stress regulation of aquaporins. Plant Mol. Biol. 59, 469–484. doi: 10.1007/s11103-005-0352-1, PMID: 16235111

[B4] Casino P. Miguel-Romero L. Marina A. (2014). Visualizing autophosphorylation in histidine kinases. Nat. Commun. 5, 3258. doi: 10.1038/ncomms4258, PMID: 24500224

[B5] Chaumont F. Moshelion M. Daniels M. J. (2005). Regulation of plant aquaporin activity. Biol. Cell 97, 749–764. doi: 10.1042/BC20040133, PMID: 16171457

[B6] Chen Q. Yang S. Kong X. Wang C. Xiang N. Yang Y.. (2018). Molecular cloning of a plasma membrane aquaporin in *Stipa purpurea*, and exploration of its role in drought stress tolerance. Gene 665, 41–48. doi: 10.1016/j.gene.2018.04.056, PMID: 29709638

[B7] Cui X. H. Hao F. S. Chen H. Chen J. Wang X. C. (2008). Expression of the *Vicia faba VfPIP1* gene in *Arabidopsis thaliana* plants improves their drought resistance. J. Plant Res. 121, 207–214. doi: 10.1007/s10265-007-0130-z, PMID: 18193401

[B8] Eisenbarth D. A. Weig A. R. (2005). Dynamics of aquaporins and water relations during hypocotyl elongation in Ricinus communis *L. seedlings* . J. Exp. Bot. 56 (417), 1831–1842. doi: 10.1093/jxb/eri173, PMID: 15897227

[B9] Gautam A. Pandey A. K. (2021). Aquaporins responses under challenging environmental conditions and abiotic stress tolerance in plants. Botanical Rev. 87, 467–495. doi: 10.1007/s12229-021-09249-z

[B10] Gill R. A. Ahmar S. Ali B. Saleem M. H. Khan M. U. Zhou W.. (2021). The role of membrane transporters in plant growth and development, and abiotic stress tolerance. Int. J. Mol. Sci. 22, 12792. doi: 10.3390/ijms222312792, PMID: 34884597 PMC8657488

[B11] Guo L. Wang Z. Y. Lin H. Cui W. E. Chen J. Liu M.. (2006). Expression and functional analysis of the rice plasma-membrane intrinsic protein gene family. Cell Res. 16, 277–286. doi: 10.1038/sj.cr.7310035, PMID: 16541126

[B12] Hoagland D. R. Arnon D. I. (1950). The water-culture method for growing plants without soil. (Berkeley: California Agricultural Experiment Station, University of California).

[B13] Horie T. Kaneko T. Sugimoto G. Sasano S. Panda S. K. Shibasaka M.. (2011). Mechanisms of water transport mediated by PIP aquaporins and their regulation via phosphorylation events under salinity stress in barley roots. Plant Cell Physiol. 52, 663–675. doi: 10.1093/pcp/pcr027, PMID: 21441236

[B14] Jang J. Y. Kim D. G. Kim Y. O. Kim J. S. Kang H. (2004). An expression analysis of a gene family encoding plasma membrane aquaporins in response to abiotic stresses in Arabidopsis thaliana. Plant Mol. Biol. 54, 713–725. doi: 10.1023/B:PLAN.0000040900.61345.a6, PMID: 15356390

[B15] Jia J. Liang Y. Hou T. Hu Y. Zhu Y. Huo H.. (2020). The expression response of plasma membrane aquaporins to salt stress in tomato plants. Environ. Exp. Bot. 178, 104190. doi: 10.1016/j.envexpbot.2020.104190

[B16] Johansson I. Larsson C. Ek B. Kjellbom P. (1996). The major integral proteins of spinach leaf plasma membranes are putative aquaporins and are phosphorylated in response to Ca^2+^ and apoplastic water potential. Plant Cell 8, 1181–1191., PMID: 8768376 10.1105/tpc.8.7.1181 PMC161200

[B17] Kaldenhoff R. Fischer M. (2006). Functional aquaporin diversity in plants. Biochim. Biophys. Acta (BBA) - Biomembranes 1758, 1134–1141. doi: 10.1016/j.bbamem.2006.03.012, PMID: 16730645

[B18] Kumar K. Mosa K. A. Meselhy A. G. Dhankher O. P. (2018). Molecular insights into the plasma membrane intrinsic proteins roles for abiotic stress and metalloids tolerance and transport in plants. Indian J. Plant Physiol. 23, 721–730. doi: 10.1007/s40502-018-0425-1

[B19] Li J. Ban L. Wen H. Wang Z. Dzyubenko N. Chapurin V.. (2015). An aquaporin protein is associated with drought stress tolerance. Biochem. Biophys. Res. Commun. 459, 208–213. doi: 10.1016/j.bbrc.2015.02.052, PMID: 25701792

[B20] Liu X. Song Y. Xing F. Wang N. Wen F. Zhu C. (2016). *Ghwrky25*, a group I WRKY gene from cotton, confers differential tolerance to abiotic and biotic stresses in transgenic Nicotiana benthamiana. Protoplasma 253, 1265–1281. doi: 10.1007/s00709-015-0885-3, PMID: 26410829

[B21] Lu K. Chen X. Yao X. An Y. Wang X. Qin L.. (2022). Phosphorylation of a wheat aquaporin at two sites enhances both plant growth and defense. Mol. Plant 15, 1772–1789. doi: 10.1016/j.molp.2022.10.003, PMID: 36207815

[B22] Maurel C. Verdoucq L. Luu D. T. Santoni V. (2008). Plant aquaporins: Membrane channels with multiple integrated functions. Annu. Rev. Plant Biol. 59, 595–624. doi: 10.1146/annurev.arplant.59.032607.092734, PMID: 18444909

[B23] Nyblom M. Frick A. Wang Y. Ekvall M. Hallgren K. Hedfalk K.. (2009). Structural and functional analysis of *SoPIP2;1* mutants adds insight into plant aquaporin gating. J. Mol. Biol. 387, 653–668. doi: 10.1016/j.jmb.2009.01.065, PMID: 19302796

[B24] Rahman A. Kawamura Y. Maeshima M. Uemura M. (2020). Plasma membrane aquaporin members PIPs act in concert to regulate cold acclimation and freezing tolerance responses in Arabidopsis thaliana. Plant Cell Physiol. 61, 787–802. doi: 10.1093/pcp/pcaa005, PMID: 31999343

[B25] Sohail H. Noor I. Nawaz M. A. Ma M. R. Shireen F. Huang Y.. (2022). Genome-wide identification of plasma-membrane intrinsic proteins in pumpkin and functional characterization of *CmoPIP1–4* under salinity stress. Environ. Exp. Bot. 202, 104995. doi: 10.1016/j.envexpbot.2022.104995

[B26] Sreedharan S. Shekhawat U. K. S. Ganapathi T. R. (2013). Transgenic banana plants overexpressing a native plasma membrane aquaporin *MusaPIP1;2* display high tolerance levels to different abiotic stresses. Plant Biotechnol. J. 11, 942–952. doi: 10.1111/pbi.12086, PMID: 23745761

[B27] Suslov M. Daminova A. Egorov J. (2024). Real-time dynamics of water transport in the roots of intact maize plants in response to water stress: The role of aquaporins and the contribution of different water transport pathways. Cells 13, 154. doi: 10.3390/cells13020154, PMID: 38247845 PMC10814095

[B28] Tang W. Page M. Fei Y. Liu L. Xu F. Cai X.. (2012). Overexpression of *AtbZIP60deltaC* gene alleviates salt-induced oxidative damage in transgenic cell cultures. Plant Mol. Biol. Rep. 30, 1183–1195. doi: 10.1007/s11105-012-0437-3

[B29] Törnroth-Horsefield S. Wang Y. Hedfalk K. Johanson U. Karlsson M. Tajkhorshid E.. (2006). Structural mechanism of plant aquaporin gating. Nature 439, 688–694. doi: 10.1038/nature04316, PMID: 16340961

[B30] Wang L. Cao H. Qian W. Yao L. Hao X. Li N.. (2017). Identification of a novel bZIP transcription factor in *Camellia sinensis* as a negative regulator of freezing tolerance in transgenic *Arabidopsis* . Ann. Bot. 119, 1195–1209. doi: 10.1093/aob/mcx011, PMID: 28334275 PMC5604549

[B31] Wang X. Gong L. Zhang J. Wang L. Wu D. Xu J. (2024). The genome-wide profiling of alternative splicing in willow under salt stress. Forests 15, 30. doi: 10.3390/f15010030

[B32] Wang L. Liu X. Gong L. Hu J. Lu X. Xu J. (2025). Overexpression of *ZsDRT102* from Zelkova schneideriana increases the chilling resistance of plants. Plant Cell Tissue Organ Culture 160, 9. doi: 10.1007/s11240-024-02947-7

[B33] Wilder V. V. Miecielica U. Degand H. Derua R. Waelkens E. Chaumont F. (2008). Maize plasma membrane aquaporins belonging to the PIP1 and PIP2 subgroups are *in vivo* phosphorylated. Plant Cell Physiol. 49, 1364–1377. doi: 10.1093/pcp/pcn112, PMID: 18682426

[B34] Yang R. Yang L. Wang X. Wang Y. Zhang J. Xu J. (2023). Over-expression of the Salix matSudana expansin gene *SmEXPA23* enhances plant salt tolerance. Plant Cell Tissue Organ Culture 152, 309–316. doi: 10.1007/s11240-022-02407-0

[B35] Yu Q. Hu Y. Li J. Wu Q. Lin Z. (2005). Sense and antisense expression of plasma membrane aquaporin BnPIP1 from Brassica napus in tobacco and its effects on plant drought resistance. Plant Sci. 169, 647–656. doi: 10.1016/j.plantsci.2005.04.013

[B36] Zhang H. Liu H. Yang R. Xu X. Liu X. Xu J. (2019). Over-expression of PttEXPA8 gene showed various resistances to diverse stresses. Int. J. Biol. Macromolecules 130, 50–57. doi: 10.1016/j.ijbiomac.2019.02.115, PMID: 30797010

[B37] Zhu C. Schraut D. Hartung W. Schaffner A. R. (2005). Differential responses of maize MIP genes to salt stress and ABA. J. Exp. Bot. 56, 2971–2981. doi: 10.1093/jxb/eri294, PMID: 16216844

